# Clinically feasible semi-automatic workflows for measuring metabolically active tumour volume in metastatic melanoma

**DOI:** 10.1007/s00259-020-05068-3

**Published:** 2020-10-25

**Authors:** Joyce van Sluis, Ellen C. de Heer, Mayke Boellaard, Mathilde Jalving, Adrienne H. Brouwers, Ronald Boellaard

**Affiliations:** 1 Department of Nuclear Medicine and Molecular Imaging, University of Groningen, University Medical Center Groningen, Hanzeplein 1, 9713GZ Groningen, The Netherlands; 2 Department of Medical Oncology, University of Groningen, University Medical Center Groningen, Hanzeplein 1, 9713 GZ Groningen, The Netherlands; 3 Department of Radiology and Nuclear Medicine, Vrije Universiteit Amsterdam, Cancer Center Amsterdam UMC, De Boelelaan 1117, 1081 HV Amsterdam, The Netherlands

**Keywords:** MATV, Quantification, Melanoma, Segmentation, FDG PET/CT

## Abstract

**Purpose:**

Metabolically active tumour volume (MATV) is a potential quantitative positron emission tomography (PET) imaging biomarker in melanoma. Accumulating data indicate that low MATV may predict increased chance of response to immunotherapy and overall survival. However, metastatic melanoma can present with numerous (small) tumour lesions, making manual tumour segmentation time-consuming. The aim of this study was to evaluate multiple semi-automatic segmentation workflows to determine reliability and reproducibility of MATV measurements in patients with metastatic melanoma.

**Methods:**

An existing cohort of 64 adult patients with histologically proven metastatic melanoma was used in this study. ^18^F-FDG PET/CT diagnostic baseline images were acquired using a European Association of Nuclear Medicine (EANM) Research Limited–accredited Siemens Biograph mCT PET/CT system (Siemens Healthineers, Knoxville, USA). PET data were analysed using manual, gradient-based segmentation and five different semi-automatic methods: three direct PET image–derived delineations (41MAX, A50P and SUV40) and two based on a majority-vote approach (MV2 and MV3), without and with (suffix ‘+’) manual lesion addition. Correlation between the different segmentation methods and their respective associations with overall survival was assessed.

**Results:**

Correlation between the MATVs derived by the manual segmentation and semi-automated tumour segmentations ranged from *R*^2^ = 0.41 for A50P to *R*^2^ = 0.85 for SUV40+ and MV2+, respectively. Manual MATV segmentation did not differ significantly from the semi-automatic methods SUV40 (∆MATV mean ± SD 0.08 ± 0.60 mL, *P* = 0.303), SUV40+ (∆MATV − 0.10 ± 0.51 mL, *P* = 0.126), MV2+ (∆MATV − 0.09 ± 0.62 mL, *P* = 0.252) and MV3+ (∆MATV − 0.03 ± 0.55 mL, *P* = 0.615). Log-rank tests showed statistically significant overall survival differences between above and below median MATV patients for all segmentation methods with areas under the ROC curves of 0.806 for manual segmentation and between 0.756 [41MAX] and 0.807 [MV3+] for semi-automatic segmentations.

**Conclusions:**

Simple and fast semi-automated FDG PET segmentation workflows yield accurate and reproducible MATV measurements that correlate well with manual segmentation in metastatic melanoma. The most readily applicable and user-friendly SUV40 method allows feasible MATV measurement in prospective multicentre studies required for validation of this potential PET imaging biomarker for clinical use.

**Electronic supplementary material:**

The online version of this article (10.1007/s00259-020-05068-3) contains supplementary material, which is available to authorized users.

## Introduction

Metastatic melanoma has evolved from being an incurable disease with notoriously poor prognosis to a cancer type with the potential of long-term survival in patients with durable responses to immunotherapy [1–5]. Despite 5-year overall survival rates of over 50% in patients with metastatic melanoma treated with a combination of anti-CTLA-4 and anti-PD1 immune checkpoint inhibitors, a substantial subset of patients does not respond [5, 6] or experiences severe side effects [5, 7–9]. Patient and tumour characteristics that are both prognostic and predictive for response to immunotherapy, such as an elevated serum lactate dehydrogenase (LDH) level and the presence of brain metastases, are far from perfect in predicting which patients will benefit [10–12]. Therefore, biomarkers to select patients or patient groups with the best chance of benefitting from these (costly) treatments are urgently needed.

High baseline (metabolically active) tumour burden is associated with worse treatment outcome and poor survival in patients with metastatic melanoma [11, 13–16]. Total body positron emission tomography (PET) using the glucose analogue 2-deoxy-2-[fluorine-18] fluoro-D-glucose (^18^F-FDG) is part of standard baseline work-up in metastatic melanoma [13]. Besides visual identification of metastases, quantitative parameters including metabolically active tumour volume (MATV) can be measured using these baseline ^18^F-FDG PET images. In patients treated with immune checkpoint inhibitors, baseline MATV was associated with survival after correction for LDH level and presence of brain metastases [11, 13, 15].

Various methods can be used to define tumour volumes of interest (VOIs), required for MATV measurements, on PET images. Manual segmentations are very labour-intensive and are prone to both intra- and interobserver variability. Consequently, semi-automated methods are being used more frequently. However, a single widely available and accepted reference method is currently lacking. The European Association of Nuclear Medicine (EANM) Research Limited (EARL) guidelines [17] recommend segmentations based on fixed standardized uptake value (SUV) VOI thresholds of 2.5 or 4.0 g/mL, 41% or 50% of the lesion’s SUVmax and 50% of the lesion’s SUVpeak adjusted for background uptake. These recommendations are mainly based on phantom studies involving uniformly filled spheres and clinical studies in non-small-cell lung carcinoma patients and patients with different types of lymphoma [18–20]. However, patients with metastatic melanoma frequently have large numbers of tumour lesions, which can be particularly small and can occur in any tissue or organ, with each organ having different background FDG uptake. These issues may hamper extrapolation of the recommended semi-automated delineation methods based on other tumour types. To our knowledge, no melanoma-specific semi-automatic segmentation studies have been published to date. For feasible large-scale evaluation of the predictive and prognostic value of quantitative PET parameters in metastatic melanoma, a fast, standardized segmentation method and corresponding workflow yielding reproducible and clinically relevant measurements is essential [21]. Recently, the need for such a standardized segmentation method to obtain MATV as a possible predictive biomarker was emphasized by E. Hindié (2020). Therefore, the aim of the current study was to develop, optimize and evaluate a clinically feasible MATV method and delineation workflow in metastatic melanoma.

## Materials and methods

### Patient population

An existing cohort of patients with metastatic melanoma (*n* = 64) was used for this study [13]. In brief, all adult patients with histologically proven cutaneous or mucosal metastatic melanoma (American Joint Committee on Cancer [AJCC] [10] 7th edition stage IV melanoma) without prior systemic treatment and with a baseline ^18^F-FDG PET/CT scan performed between May 2014 and December 2015 with PET-positive lesions were included in the cohort. Patients also underwent a baseline contrast-enhanced diagnostic CT scan around the time of PET/CT scanning [22]. Patient and tumour characteristics were retrieved retrospectively from the electronic patient files (see Table 1 in [13] or Supplemental Table 1 for a modified version).

**Table 1:** Overview of the various tumour delineation methods

Segmentation method	VOI delineation threshold
Manual	Visual, gradient-based
Semi-automatic	
SUV40	SUV = 4.0 g/mL
41MAX	41% lesion SUVmax
A50P	50% lesion SUVpeak, corrected for background
MV2	Voxels included by ≥ 2 of SUV25, SUV40, 41MAX and A50P
MV3	Voxels included by ≥ 3 of SUV25, SUV40, 41MAX and A50P

The local medical ethics committee approved the study and the need for written informed consent for this retrospective analysis was waived (case number: 2016/474). The institutional objection registry indicated that the selected patients had not objected to the use of their personal data for research purposes. Patient data and images were pseudonymized, and data were stored on a secured server according to local data management regulations.

### Imaging protocol

Baseline ^18^F-FDG PET scans were acquired using an EARL accredited Siemens Biograph mCT PET/CT system (Siemens Healthineers, Knoxville, USA). PET image acquisition was performed according to EANM guidelines for tumour imaging [17]. Acquired images were reconstructed using 3D TOF OP-OSEM with 3 iterations and 21 subsets, and a Gaussian filter of 6.5 mm into an image matrix size of 256 × 256 with a voxel size of 3.2 × 3.2 × 2 mm. Patients were instructed to fast and avoid exercise at least 4–6 h prior to intravenous ^18^F-FDG injection (3 MBq/kg activity). Plasma glucose levels were < 198 mg/dL before ^18^F-FDG administration and the time interval between ^18^F-FDG injection and imaging was 60 min (± 5 min). Total body PET imaging (from the top of the head to and including the feet) was conducted with 1–3 min per bed position (depending on body weight). Prior to PET acquisition, patients underwent a low-dose CT (non-contrast-enhanced) for attenuation and scatter correction (tube voltage of 80–140 kV, tube current of 30 mAs and a spiral pitch factor of 1).

### Image analysis

PET images were analysed using ACCURATE, an in-house developed image analysis tool [23]. PET data of all 64 patients had previously been delineated using a manual, gradient-based segmentation method as described in [13] (observer 1). For this study, using the same gradient-based segmentation method, PET images of the first 20 patients were delineated by a second observer (observer 2) to determine interobserver variability in manual MATV measurements. Furthermore, PET data of all 64 patients were analysed using six different semi-automatic segmentation methods and corresponding workflows.

The total tumour burden (TTB) tool in ACCURATE is based on four commonly used PET image–based segmentation methods [17, 18, 24–26]. The different methods have been described previously by Kolinger et al. In short, the PET image–based segmentation methods are as follows: a fixed SUV threshold of 2.5 g/mL (SUV25), a fixed SUV threshold of 4.0 g/mL (SUV40), an adaptive threshold at 41% of each lesion’s SUVmax (41MAX) and a contrast corrected threshold for local tumour-to-background activity at 50% of the lesion’s SUVpeak (A50P). SUV25 was not included as an individual segmentation method in the final study because the first cases analysed by this approach resulted in VOIs that included large areas of healthy tissue requiring substantial manual corrections. Therefore, we did not consider this method to be clinically feasible and omitted it in the analysis of the remaining scans. In addition, two consensus methods, so-called majority-vote methods, are available in the TTB tool: agreement between two or more of the four abovementioned standard PET-based methods (MV2) and agreement between three or more of the four standard PET-based methods (MV3) (Table 1) [27]. Furthermore, the TTB tool requires a minimal lesion volume which was set to 3 mL for all methods in the current study.

The TTB tool yields automatically segmented VOIs of all areas fulfilling the abovementioned thresholds. Regions with physiologically high uptake (such as the bladder, kidneys, the myocardium and the brain) can be removed manually by a single mouse click. Subsequently, all individual VOIs are saved and summed (referred to as VOI_total_) and used to derive quantitative image parameters. Optionally, all lesions initially overlooked by the thresholding algorithm can be selected and added to the total VOI by the observer using single mouse clicks. Addition of all visible lesions to the VOI may increase MATV accuracy as indicated in a lymphoma study [26]. When all visible lesions have been added, the final summed VOI is saved again (referred to as VOI_total_+) and also used to derive quantitative imaging parameters.

For comparison and completeness, we additionally explored the prognostic value of other PET biomarkers: SUVmax, SUVpeak and TLG.

### Statistical analysis

Statistical analyses were performed using SPSS Statistics, version 25.0 (IBM Corp., Armonk, NY) and Rstudio version 1.1.463. Normal distribution of the data was assessed using Q-Q plots. Interobserver agreement between the MATV measurements obtained through manual VOI segmentation was analysed using Pearson’s correlation analysis, relative difference plots and boxplots. Correlation between MATVs obtained from manual and semi-automatic methods and among MATVs obtained from different semi-automatic methods was assessed using Pearson’s correlation analysis. Differences between the MATVs obtained using the different semi-automatic methods, and between the MATVs of the manual segmentation versus the different semi-automatic methods, were explored using paired samples *t* tests after log transformation of the data. A *P* value of less than 0.05 was considered significant. For quantification of VOI similarity, the Jaccard similarity coefficient and overlap fraction between manually segmented VOIs of observer 1 and VOIs obtained using each semi-automated segmentation method were calculated.

For each segmentation method, receiver-operating-characteristic (ROC) curves were obtained to assess associations of the differently obtained MATVs with overall survival. Kaplan-Meier plots were used to estimate overall survival (defined as time between baseline PET and date of death or last follow-up). Patients were stratified into two groups based on the median MATV for each segmentation method and log-rank tests were performed to test whether these groups had significantly different survival curves.

## Results

### Manual segmentation versus TTB tool

**Fig. 1:**
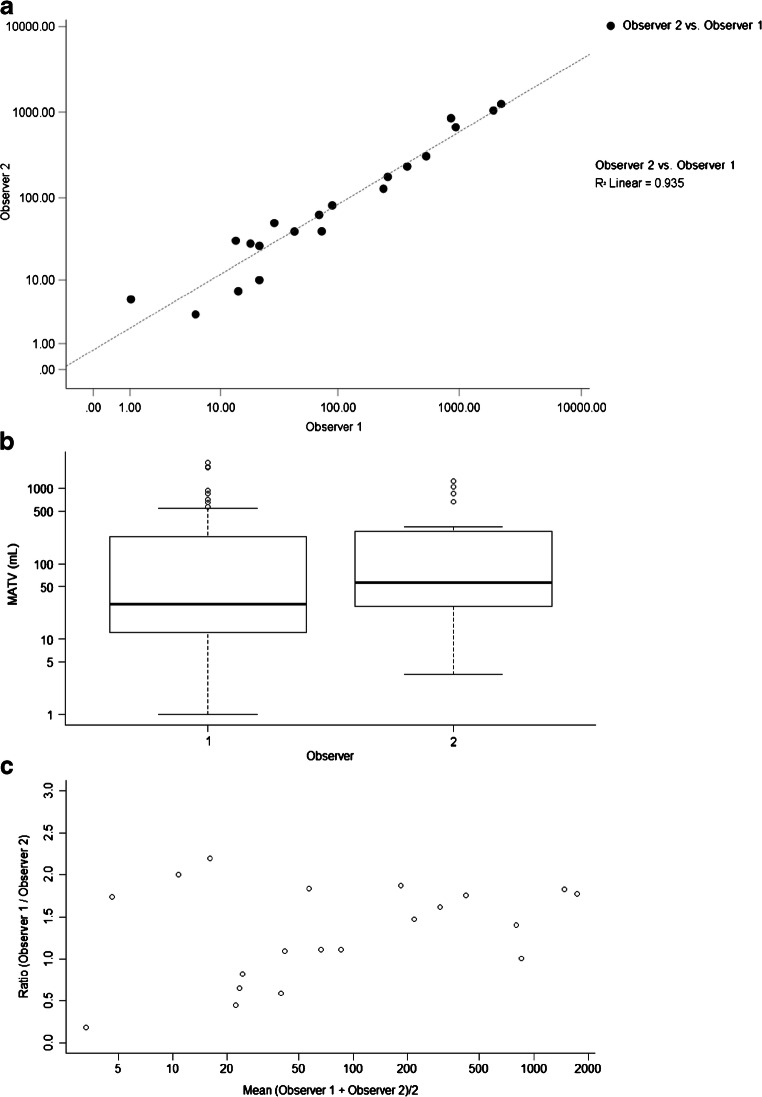
Scatter plot (**a**), boxplots (**b**) and ratio plot (**c**) of MATV measurements (mL) obtained through manual VOI segmentation of the first 20 patients (*n* = 20) delineated by observer 1 and observer 2. The dashed line in **a** indicates the regression between the measurements of both observers

**Fig. 2:**
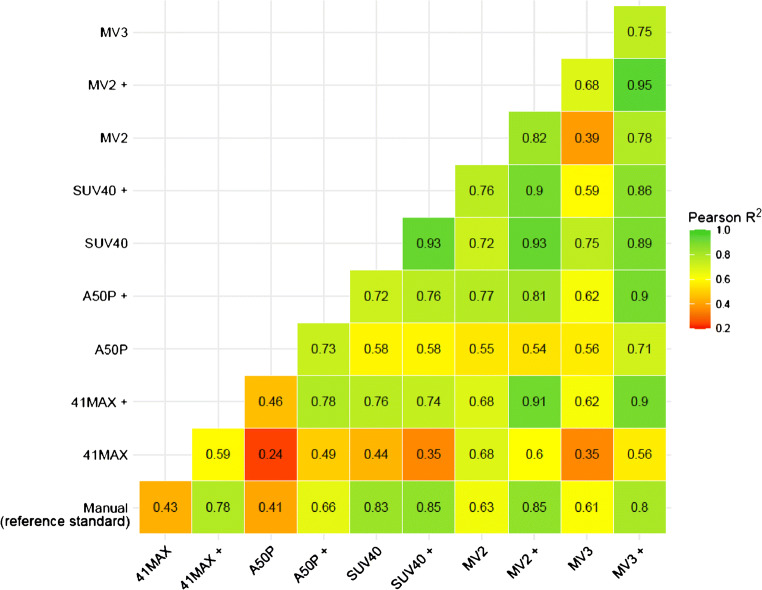
Correlogram between the MATV measurements (*n* = 64) obtained from the manual VOI segmentations of observer 1 and the MATV measurements obtained through use of semi-automated segmentation in the TTB tool without additional lesion selection, i.e. VOI_total_, and with additional lesion selection, i.e. VOI_total_+. 41MAX, the semi-automated segmentation method using 41% of the lesion’s SUVmax; A50P, the semi-automated segmentation method using 50% of the lesion’s SUVpeak adjusted for background uptake; SUV40, the semi-automated segmentation method using a fixed SUV threshold of 4.0 g/mL; MV2, consensus “majority-vote” method using agreement between 2 or more of the standard PET-based methods; MV3, consensus “majority-vote” method using agreement between 3 or more of the standard PET-based methods

**Fig. 3:**
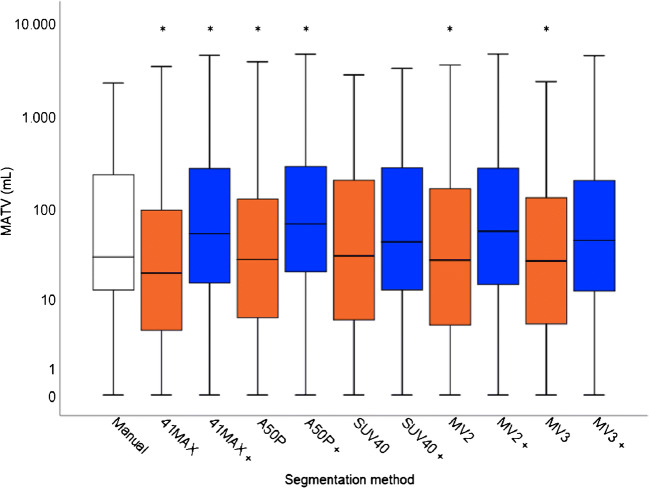
Boxplots showing the spread of MATV obtained through manual VOI segmentations of observer 1 (white) and all semi-automatic segmentations (*n* = 64) without (orange), i.e. VOI_total_, and with (blue) additional lesion inclusion, i.e. VOI_total_+. The boxes bound the interquartile range (IQR) divided by the median MATV (indicated by the thick horizontal black line). The whiskers extend to a maximum of 1.5*IQR beyond the box. The asterisks indicate a significant difference (*P* < 0.05) between the MATV measurements obtained using the semi-automated methods and the manual segmentation. Please note, in some patients, semi-automatic segmentation methods do not succeed in capturing any lesions (for example in cases of lesions < 3 mL). In these cases, MATV equals zero. 41MAX, the semi-automated segmentation method using 41% of the lesion’s SUVmax; A50P, the semi-automated segmentation method using 50% of the lesion’s SUVpeak adjusted for background uptake; SUV40, the semi-automated segmentation method using a fixed SUV threshold of 4.0 g/mL; MV2, consensus “majority-vote” method using agreement between 2 or more of the standard PET-based methods; MV3, consensus “majority-vote” method using agreement between 3 or more of the standard PET-based methods

A high interobserver correlation was found between the manual segmentation in the first 20 patients (*R*^2^ = 0.935) (Fig. 1). Figure 1b shows boxplots of the MATVs of both observers demonstrating a good agreement between both observers. Manually derived MATVs were similar between observer 1 and observer 2 (*n* = 20; *P* = 0.314, ∆MATV mean ± SD 0.06 ± 0.27 mL, 95% CI [− 0.06–0.19]). Correlations between the manual segmentations by observer 1 and the five different semi-automatic segmentation methods (with, i.e. VOI_total_+, and without additional lesion selection, i.e. VOI_total_) ranged from *R*^2^ = 0.41 to *R*^2^ = 0.85 (Figs. 2 and 3). MATVs were equal to zero in cases where the semi-automatic segmentation method was not able to segment any voxels, e.g. when none of the voxels exceeded the fixed SUV and/or volumetric segmentation thresholds.

When observers were allowed to select additional lesions that were initially not included in the automated preselection (VOI_total_+), total summed MATV increased by 166%, 86%, 18%, 53% and 89% for the 41MAX, A50P, SUV40, MV2 and MV3 methods, respectively.

Log transformed MATV derived by manual segmentation did not differ between observer 1 and the semi-automatic SUV40 method (∆MATV mean ± SD 0.08 ± 0.60 mL, 95% CI [− 0.07–0.23], *P* = 0.303); the semi-automatic SUV40+ method (∆MATV mean ± SD − 0.10 ± 0.51 mL, 95% CI [− 0.23–0.03], *P* = 0.126); the semi-automatic MV2+ method (∆MATV mean ± SD − 0.09 ± 0.62 mL, 95% CI [− 0.24–0.06], *P* = 0.252); or the semi-automatic MV3+ method (∆MATV mean ± SD − 0.03 ± 0.55 mL, 95% CI [− 0.17–0.10], *P* = 0.615). All other semi-automated segmentation methods (VOI_total_ and VOI_total_+) showed significant differences in MATVs values derived compared to manual segmentation by observer 1 (*P*≤ 0.05).

**Fig. 4:**
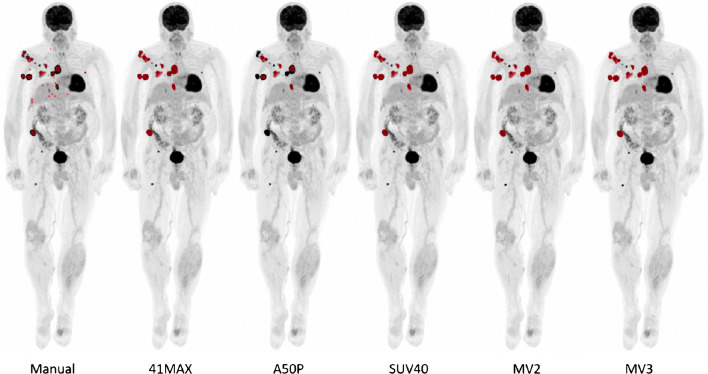
Example MIP images of a single patient for comparison of the manual segmentations of observer 1 and all semi-automated segmentations without additional lesion selection. 41MAX, the semi-automated segmentation method using 41% of the lesion’s SUVmax; A50P, the semi-automated segmentation method using 50% of the lesion’s SUVpeak adjusted for background uptake; SUV40, the semi-automated segmentation method using a fixed SUV threshold of 4.0 g/mL; MV2, consensus “majority-vote” method using agreement between 2 or more of the standard PET-based methods; MV3, consensus “majority-vote” method using agreement between 3 or more of the standard PET-based methods

**Fig. 5:**
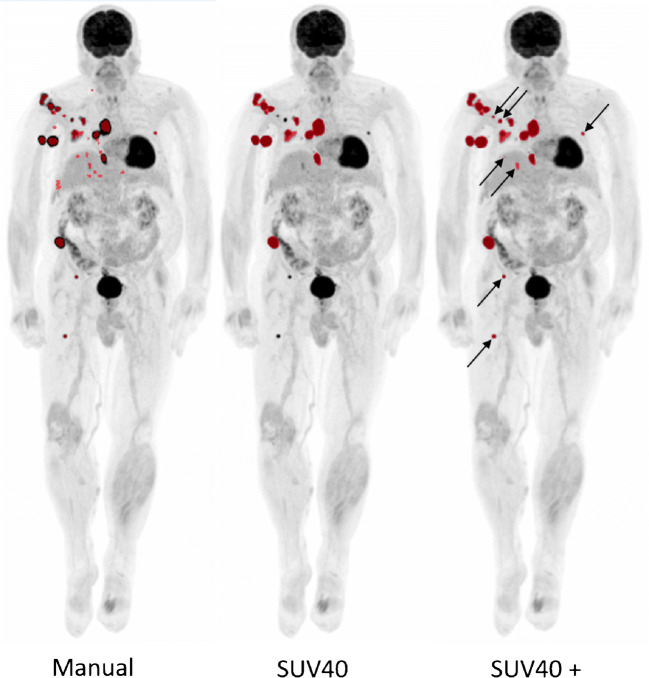
Example MIP images of a single patient for comparison of the manual segmentations of observer 1 (left) and the semi-automated SUV40 segmentations without additional lesion selection (middle) and the semi-automated SUV40+ segmentations with additional lesion selection (right). Arrows indicate manually added lesions. SUV40, the semi-automated segmentation method using a fixed SUV threshold of 4.0 g/mL

The Jaccard similarity coefficient and overlap fraction were determined to quantify overlap between manually segmented VOIs and the VOIs obtained through semi-automated segmentation (Table 2). For illustrative purposes, example MIP images showing the manual segmentations of observer 1 versus the VOIs obtained with semi-automated segmentation methods with and without additional lesion selection are shown in Figs. 4 and 5, respectively.

**Table 2:** Jaccard similarity coefficient and percentage overlap between VOIs. All semi-automated segmentation methods were compared to manual delineations by observer 1. Please note, in some patients, semi-automatic segmentation methods did not succeed in capturing any lesions (for example in cases of lesions < 3 mL) in contrast to the manual delineation. In these cases, semi-automatic MATV and, consequently, the Jaccard coefficient and overlap fraction equal zero

Segmentation method	Jaccard coefficient (mean ± SD, range)	Fraction overlap (mean ± SD, range)
41MAX	0.29 ± 0.26, 0–0.79	0.34 ± 0.29, 0–0.95
41MAX+	0.44 ± 0.25, 0–0.87	0.65 ± 0.29, 0–1
A50P	0.30 ± 0.25, 0–0.78	0.38 ± 0.30, 0–0.89
A50P+	0.38 ± 0.23, 0–0.94	0.64 ± 0.30, 0–1
SUV40	0.30 ± 0.24, 0–0.79	0.46 ± 0.35, 0–1
SUV40+	0.39 ± 0.21, 0–0.83	0.62 ± 0.32, 0–1
MV2	0.27 ± 0.23, 0–0.83	0.42 ± 0.34, 0–0.98
MV2+	0.32 ± 0.24, 0–0.86	0.59 ± 0.38, 0–1
MV3	0.30 ± 0.26, 0–0.80	0.38 ± 0.30, 0–0.87
MV3+	0.38 ± 0.26, 0–0.85	0.58 ± 0.35, 0–0.98

### Survival

**Fig. 6:**
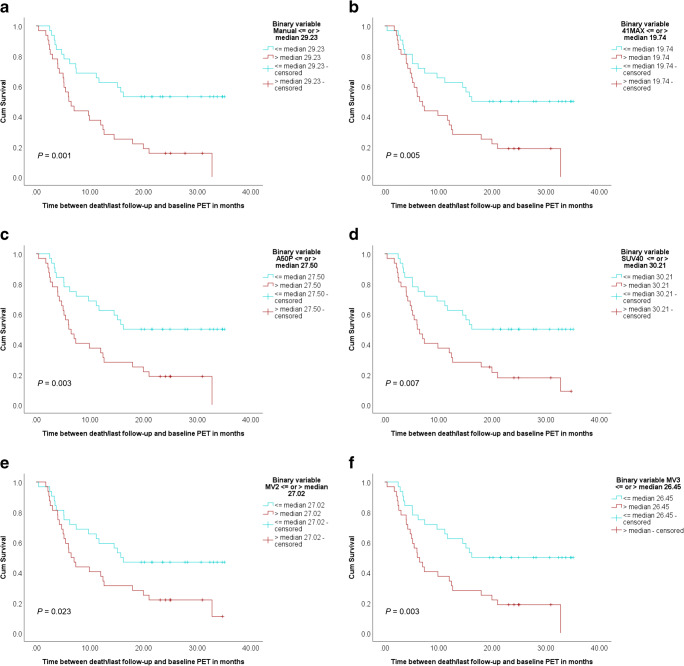
Kaplan-Meier curves and log-rank test *P* values for overall survival of all patients (*n* = 64) based on median MATV obtained through manual segmentation (**a**) and semi-automatic segmentation without additional lesion selection using the different quantitative PET image–based thresholds incorporated in the TTB tool: 41MAX (**b**), A50P (**c**), SUV40 (**d**), MV2 (**e**) and MV3 (**f**). 41MAX, the semi-automated segmentation method using 41% of the lesion’s SUVmax; A50P, the semi-automated segmentation method using 50% of the lesion’s SUVpeak adjusted for background uptake; SUV40, the semi-automated segmentation method using a fixed SUV threshold of 4.0 g/mL; MV2, consensus “majority-vote” method using agreement between 2 or more of the standard PET-based methods; MV3, consensus “majority-vote” method using agreement between 3 or more of the standard PET-based methods

**Fig. 7:**
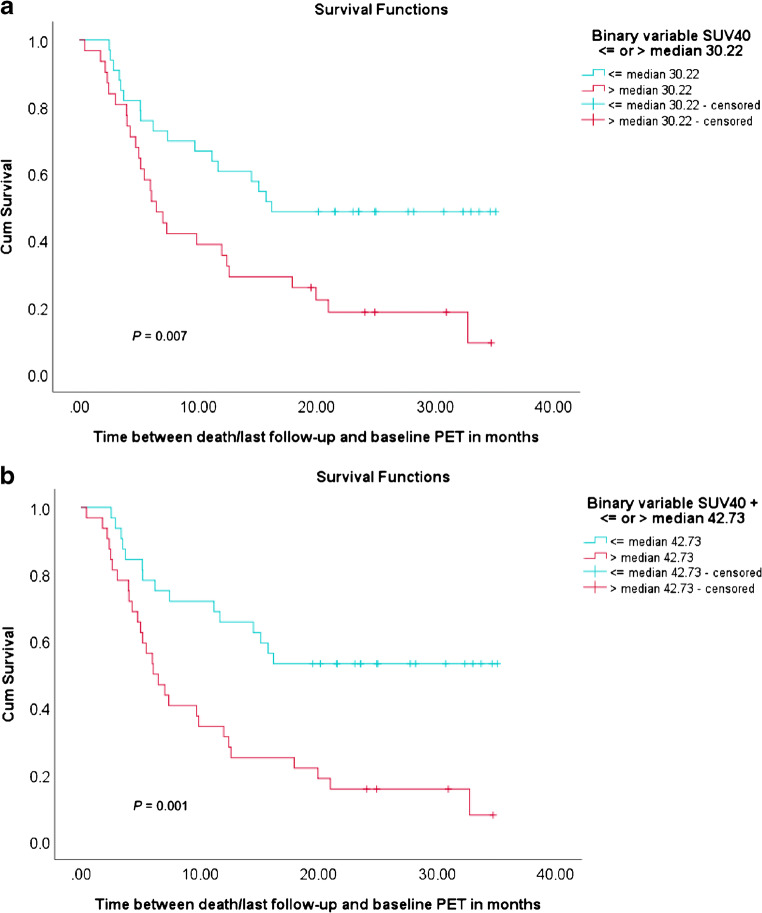
Kaplan-Meier curves and log-rank test *P* values for overall survival of all patients (*n* = 64) based on median MATV obtained through semi-automatic VOI_total_ segmentation without additional lesion selection (**a**), i.e. VOI, and with additional lesion selection (**b**), i.e. VOI_total_+, using the quantitative PET image–based SUV40 method. SUV40, the semi-automated segmentation method using a fixed SUV threshold of 4.0 g/mL

**Fig. 8:**
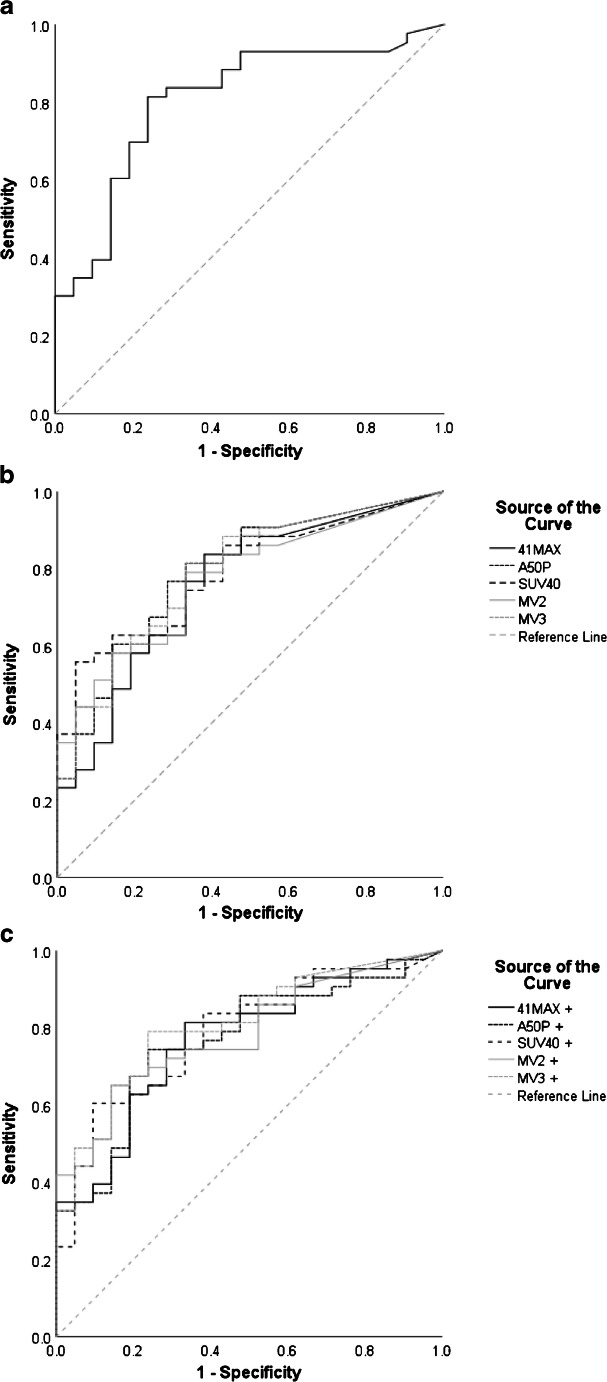
ROC curves assessing survival predictability based on MATV measurement by manual VOI segmentation (**a**), semi-automatic VOI segmentation, i.e. VOI_total_ (**b**), and semi-automatic VOI segmentation with additional lesion selection, i.e. VOI_total_+ (**c**). 41MAX, the semi-automated segmentation method using 41% of the lesion’s SUVmax; A50P, the semi-automated segmentation method using 50% of the lesion’s SUVpeak adjusted for background uptake; SUV40, the semi-automated segmentation method using a fixed SUV threshold of 4.0 g/mL; MV2, consensus “majority-vote” method using agreement between 2 or more of the standard PET-based methods; MV3, consensus “majority-vote” method using agreement between 3 or more of the standard PET-based methods

**Fig. 9:**
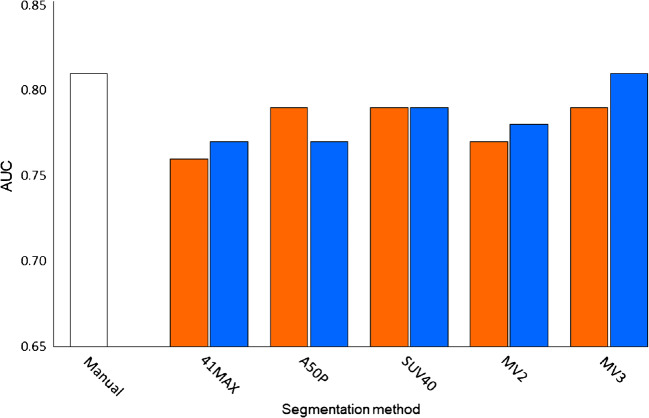
Bar plot of the AUC of the ROC curves illustrating the difference in accuracy of predicting survival outcome based on MATV obtained using manual tumour segmentations (white) and through use of semi-automatic segmentation methods (without (orange), i.e. VOI_total_, and with additional lesion selection (blue)), i.e. VOI_total_+. 41MAX, the semi-automated segmentation method using 41% of the lesion’s SUVmax; A50P, the semi-automated segmentation method using 50% of the lesion’s SUVpeak adjusted for background uptake; SUV40, the semi-automated segmentation method using a fixed SUV threshold of 4.0 g/mL; MV2, consensus “majority-vote” method using agreement between 2 or more of the standard PET-based methods; MV3, consensus “majority-vote” method using agreement between 3 or more of the standard PET-based methods

At the time of analysis (17.9 months after the last baseline PET acquisition), 21 of the included 64 patients (32.8%) were still alive. Patients (*n* = 64) were divided into two groups, a high and a low MATV group, for each segmentation method based on the median MATV. Kaplan-Meier curves for overall survival showed good separation of the high and low MATV curves and were statistically significant (*P* < 0.05) for each of the semi-automatic segmentation methods (without additional lesion selection) (Fig. 6b–f) as has been described previously for the manual method (Fig. 6a). Selecting additional lesions (i.e. VOI_total_+) did not improve the association of overall survival with MATV compared to automatic segmentation without selecting additional lesions (i.e. VOI_total_) (Fig. 7). The ROC curves reveal no significant differences regarding sensitivity and specificity for predicting overall survival, with similar areas under the curves (Figs. 8 and 9, Table 3). Kaplan-Meier survival curves were similar for the other quantitative FDG PET parameters SUVmax, SUVpeak and/or total lesion glycolysis (TLG) compared to MATV (see Supplemental Fig. 1). Corresponding ROC curves with associated areas under the curves were slightly higher for (combinations with) MATV and its associated parameter TLG than the independent biomarker SUVmax or SUVpeak (see Supplemental Fig. 2 and Supplemental Table 2).

**Table 3:** Areas under the ROC curves (see Fig. 5). A comparison can be made between manual segmentation, semi-automatic VOI segmentation (i.e. VOI_total_), and semi-automatic VOI segmentation with additional lesion selection (i.e. VOI_total_+). 41MAX, the semi-automated segmentation method using 41% of the lesion’s SUVmax; A50P, the semi-automated segmentation method using 50% of the lesion’s SUVpeak adjusted for background uptake; SUV40, the semi-automated segmentation method using a fixed SUV threshold of 4.0 g/mL; MV2, consensus “majority-vote” method using agreement between 2 or more of the standard PET-based methods; MV3, consensus “majority-vote” method using agreement between 3 or more of the standard PET-based methods

Segmentation method			Area under the ROC curve
Manual	VOI	VOI+	
Observer 1			0.806
	41MAX		0.756
	A50P		0.791
	SUV40		0.790
	MV2		0.772
	MV3		0.790
		41MAX+	0.770
		A50P+	0.766
		SUV40+	0.785
		MV2+	0.783
		MV3+	0.807

## Discussion

This study shows that MATV measurements by rapid semi-automatic segmentation methods correlate well with MATVs derived by manual tumour delineation. MATV is a quantitative FDG PET biomarker with potential prognostic and/or predictive value in patients with metastatic melanoma and these rapid semi-automatic segmentation methods make further clinical validation of this biomarker feasible [25, 28].

We found a high interobserver correlation between manual, gradient-based MATV delineations in metastatic melanoma patients. Furthermore, manually obtained MATVs correlated well with five different semi-automatic segmentation methods. The time expenditure for the different segmentation methods is highly variable. Difference in time expenditure could play an important role in determining the most suitable segmentation method for future use. Manual segmentation of all lesions took more than 1 day for several of the patients studied (data not shown), depending on the number of small metastases. A similar retrospective study evaluating FDG PET parameters in melanoma patients (*n* = 56) used manual lesion indication followed by semi-automatic contouring with a 40% SUVmax threshold [15] and reported an average delineation time per patient of 10 min. The shorter time expenditure reported might be explained by differences in the extent of manual delineation (complete lesion delineation vs. only manual lesion indication in [15]) and the inclusion of patients with stage III, i.e. with less widespread disease, as opposed to only stage IV patients in our cohort. Using the automated methods presented in our paper, segmentations always finished in ~3 min. When additionally selecting initially missed but visible lesions, total processing time per patient increased to 10–30 min (depending on the number of metastases and the PET segmentation threshold settings). However, including all lesions by manually adding initially missed ones (i.e. VOI_total_+) did not change the association of MATV with survival. This implies that the fast and simple workflow of semi-automated segmentation method works equally well as time-consuming and error-prone manual delineation, even when excluding lesions < 3 mL. This justifies omitting manual interference with the semi-automatic methods when evaluating associations of MATV with survival, since this provides the most time-efficient and observer-independent measurement. The lack of additional benefit of adding initially missed lesions also emphasizes the robustness of MATV as a potential PET biomarker to predict overall survival in metastatic melanoma.

The excellent performance of semi-automated MATV measurements compared to manual segmentation was also found in other studies evaluating MATV for survival prediction in different cancer types [19, 20, 28]. In patients with diffuse large B cell lymphoma, MATV was measured using two types of quantitative PET image analysis software. This software incorporated three different PET image–based thresholds (a SUVmax of 2.5 g/mL, 41MAX, and all voxels > SUVmean in a spherical VOI of 3 cm^3^ placed in the liver as recommended in the PERCIST guidelines (PERCIST Hermes [29]). Depending on the delineation method, different MATVs were obtained but all methods predicted survival outcome with similar accuracy [19]. Likewise, the strong prognostic value of MATV for survival outcome in peripheral T cell lymphoma was similar for four different PET image–based adaptive thresholding methods (signal-to-background ratio, tumour-to-background intensities, 3-dimensional geometric model based on spatial resolution and mean SUVmax) [20].

The absolute fixed threshold SUV40 was the semi-automatic MATV measurement method that best correlated with manual segmentation. For this method, selection of additional lesions (VOI_total_+) had the least impact on the MATV and did not improve the AUC of the ROC curve. Moreover, the SUV40 method was the least time-consuming and was perceived as the most user-friendly method. Therefore, use of the semi-automated SUV40 method without additional lesion selection is recommended for further studies.

Limitations of the current study include its retrospective nature and heterogeneity in patient treatments. Although treatments do not influence baseline MATV measurements or correlations among the segmentation methods, the associations with survival might change. Furthermore, in the standard of care PET/CT acquisition a non-contrast-enhanced low-dose CT is obtained, which has a lower sensitivity than PET combined with contrast-enhanced CT for most metastatic locations [22]. Although this can have implications for lesion detection and management in specific clinical cases, it is highly unlikely that the PET-based MATV segmentations will change by non-contrast-enhanced vs. contrast-enhanced CT. Moreover, previous assessment of the contrast-enhanced CT in a subset of the cohort revealed only 0.4% (small) additional FDG PET-negative lesions [13]. Additionally, since all measurements were performed on ^18^F-FDG PET images only, brain metastases (observed in 22 patients) could not be included in the automated MATV measurements. However, the contribution of brain metastasis to the total MATV is generally small [30] and individual brain metastases are often < 1 mL based on MRI [31, 32], i.e. below the used threshold of 3 mL lesion volume in the TTB.

In summary, we found that a semi-automated segmentation workflow, especially using the SUV40 method, provides a fast and robust approach for measuring MATV in melanoma patients. The association of MATV with overall survival was similar for semi-automated methods compared to manual delineation. The proposed workflow is a promising, clinically feasible approach for measuring MATV and is a good starting point for prospective multicentre validation of MATV as quantitative (predictive and/or prognostic) imaging biomarker in melanoma patients.

## Conclusion

In metastatic melanoma patients, the quantitative imaging biomarker MATV can be obtained using the robust, rapid and simple semi-automated SUV40 segmentation approach. This straightforward approach allows measurement of MATV in large prospective multicentre studies required for validation of this FDG PET imaging parameter as a predictive and/or prognostic biomarker in the clinic.

## Electronic supplementary material

ESM 1 (DOCX 200 kb)

## References

[1] Long GV, Flaherty KT, Stroyakovskiy D, Gogas H, Levchenko E, de Braud F (2019). Dabrafenib plus trametinib versus dabrafenib monotherapy in patients with metastatic BRAF V600E/K-mutant melanoma: long-term survival and safety analysis of a phase 3 study. Ann Oncol.

[2] Knispel S, Zimmer L, Kanaki T, Ugurel S, Schadendorf D, Livingstone E (2018). The safety and efficacy of dabrafenib and trametinib for the treatment of melanoma. Expert Opin Drug Saf.

[3] Andor N, Graham TA, Jansen M, Xia LC, Aktipis CA, Petritsch C (2016). Pan-cancer analysis of the extent and consequences of intratumor heterogeneity. Nat Med.

[4] Ugurel S, Röhmel J, Ascierto PA, Flaherty KT, Grob JJ, Hauschild A (2017). Survival of patients with advanced metastatic melanoma: the impact of novel therapies–update 2017. Eur J Cancer.

[5] Hindié E. Metastatic melanoma: can FDG-PET predict success of anti-PD-1 therapy and help determine when it can be discontinued? Eur J Nucl Med Mol Imaging. 2020:2–7. 10.1007/s00259-020-04826-7 32322914

[6] Larkin J, Chiarion-Sileni V, Gonzalez R, Grob J-J, Rutkowski P, Lao CD (2019). Five-year survival with combined nivolumab and ipilimumab in advanced melanoma. N Engl J Med.

[7] Daud A, Tsai K (2017). Management of treatment-related adverse events with agents targeting the MAPK pathway in patients with metastatic melanoma. Oncologist..

[8] Blank CU, Larkin J, Arance AM, Hauschild A, Queirolo P, Del Vecchio M (2017). Open-label, multicentre safety study of vemurafenib in 3219 patients with BRAF V600 mutation-positive metastatic melanoma: 2-year follow-up data and long-term responders’ analysis. Eur J Cancer.

[9] Hodi FS, Chiarion-Sileni V, Gonzalez R, Grob JJ, Rutkowski P, Cowey CL (2018). Nivolumab plus ipilimumab or nivolumab alone versus ipilimumab alone in advanced melanoma (CheckMate 067): 4-year outcomes of a multicentre, randomised, phase 3 trial. Lancet Oncol.

[10] Balch CM, Gershenwald JE, Soong SJ, Thompson JF, Atkins MB, Byrd DR (2009). Final version of 2009 AJCC melanoma staging and classification. J Clin Oncol.

[11] Ito K, Schöder H, Teng R, Humm JL, Ni A, Wolchok JD (2019). Prognostic value of baseline metabolic tumor volume measured on 18 F-fluorodeoxyglucose positron emission tomography/computed tomography in melanoma patients treated with ipilimumab therapy. Eur J Nucl Med Mol Imaging.

[12] Luke JJ, Flaherty KT, Ribas A, Long GV (2017). Targeted agents and immunotherapies: optimizing outcomes in melanoma. Nat Rev Clin Oncol.

[13] De Heer EC, Brouwers AH, Boellaard R, Sluiter WJ, Diercks GFH, Hospers GAP, et al. Mapping heterogeneity in glucose uptake in metastatic melanoma using quantitative ^18^F-FDG PET / CT analysis. EJNMMI Res. 2018;8:101. 10.1186/s13550-018-0453-x PMC6246760 30460579

[14] Joseph RW, Elassaiss-Schaap J, Kefford R, Hwu WJ, Wolchok JD, Joshua AM (2018). Baseline tumor size is an independent prognostic factor for overall survival in patients with melanoma treated with pembrolizumab. Clin Cancer Res.

[15] Seban R-D, Moya-Plana A, Antonios L, Yeh R, Marabelle A, Deutsch E (2020). Prognostic 18F-FDG PET biomarkers in metastatic mucosal and cutaneous melanoma treated with immune checkpoint inhibitors targeting PD-1 and CTLA-4. Eur J Nucl Med Mol Imaging.

[16] Reinert CP, Gatidis S, Sekler J, Dittmann H, Pfannenberg C, La Fougère C (2020). Clinical and prognostic value of tumor volumetric parameters in melanoma patients undergoing 18F-FDG-PET/CT: a comparison with serologic markers of tumor burden and inflammation. Cancer Imaging.

[17] Boellaard R, Delgado-Bolton R, Oyen WJG, Giammarile F, Tatsch K, Eschner W (2014). FDG PET/CT: EANM procedure guidelines for tumour imaging: version 2.0. Eur J Nucl Med Mol Imaging.

[18] Frings V, de Langen AJ, Smit EF, van Velden FHP, Hoekstra OS, van Tinteren H (2010). Repeatability of metabolically active volume measurements with 18F-FDG and 18F-FLT PET in non-small cell lung cancer. J Nucl Med.

[19] Ilyas H, Mikhaeel NG, Dunn JT, Rahman F, Møller H, Smith D (2018). Defining the optimal method for measuring baseline metabolic tumour volume in diffuse large B cell lymphoma. Eur J Nucl Med Mol Imaging.

[20] Cottereau A-S, Hapdey S, Chartier L, Modzelewski R, Casasnovas O, Itti E (2017). Baseline total metabolic tumor volume measured with fixed or different adaptive thresholding methods equally predicts outcome in peripheral T cell lymphoma. J Nucl Med.

[21] O’Connor JPB, Aboagye EO, Adams JE, Aerts HJWL, Barrington SF, Beer AJ (2017). Imaging biomarker roadmap for cancer studies. Nat Rev Clin Oncol.

[22] Bisschop C, de Heer EC, Brouwers AH, Hospers GAP, Jalving M (2020). Rational use of 18F-FDG PET/CT in patients with advanced cutaneous melanoma: a systematic review. Crit Rev Oncol Hematol.

[23] Boellaard R. Quantitative oncology molecular analysis suite: ACCURATE. J Nucl Med. Society of Nuclear Medicine; 2018;59:1753–1753. Available from: http://jnm.snmjournals.org/cgi/content/short/59/supplement_1/1753. Accessed on 20 December 2019.

[24] van Velden FHP, Kramer GM, Frings V, Nissen IA, Mulder ER, de Langen AJ (2016). Repeatability of radiomic features in non-small-cell lung cancer [18F]FDG-PET/CT studies: impact of reconstruction and delineation. Mol Imaging Biol.

[25] Cheebsumon P, Yaqub M, Van Velden FHP, Hoekstra OS, Lammertsma AA, Boellaard R (2011). Impact of [ 18F] FDG PET imaging parameters on automatic tumour delineation: need for improved tumour delineation methodology. Eur J Nucl Med Mol Imaging.

[26] Burggraaff CN, Rahman F, Kaßner I, Pieplenbosch S, Barrington SF, Jauw YWS (2020). Optimizing workflows for fast and reliable metabolic tumor volume measurements in diffuse large B cell lymphoma. Mol Imaging Biol.

[27] Kolinger GD, Vállez García D, Kramer GM, Frings V, Smit EF, de Langen AJ (2019). Repeatability of [ 18 F] FDG PET/CT total metabolic active tumour volume and total tumour burden in NSCLC patients. EJNMMI Res.

[28] Im HJ, Bradshaw T, Solaiyappan M, Cho SY (2018). Current methods to define metabolic tumor volume in positron emission tomography: which one is better?. Nucl Med Mol Imaging.

[29] Wahl RL, Jacene H, Kasamon Y, Lodge MA (2009). From RECIST to PERCIST: evolving considerations for PET response criteria in solid tumors. J Nucl Med.

[30] Hirshman BR, Wilson BR, Ali MA, Schupper AJ, Proudfoot JA, Goetsch SJ (2018). Cumulative intracranial tumor volume augments the prognostic value of the diagnosis-specific graded prognostic assessment model for survival in patients with melanoma cerebral metastases. Clin Neurosurg.

[31] Hadi I, Roengvoraphoj O, Bodensohn R, Hofmaier J, Niyazi M, Belka C (2020). Stereotactic radiosurgery combined with targeted/ immunotherapy in patients with melanoma brain metastasis. Radiat Oncol.

[32] Ahmed KA, Stallworth DG, Kim Y, Johnstone PAS, Harrison LB, Caudell JJ (2016). Clinical outcomes of melanoma brain metastases treated with stereotactic radiation and anti-PD-1 therapy. Ann Oncol.

