# Supplementary material for: Clinically feasible semi-automatic workflows for measuring metabolically active tumour volume in metastatic melanoma
Source: Eur J Nucl Med Mol Imaging. 2020 Oct 25;48(5):1498–510. doi: 10.1007/s00259-020-05068-3 (PMC8113298; doi:10.1007/s00259-020-05068-3)
Supplement: Supplementary file 1 — (DOCX 200 kb) [file 259_2020_5068_MOESM1_ESM.docx]

***
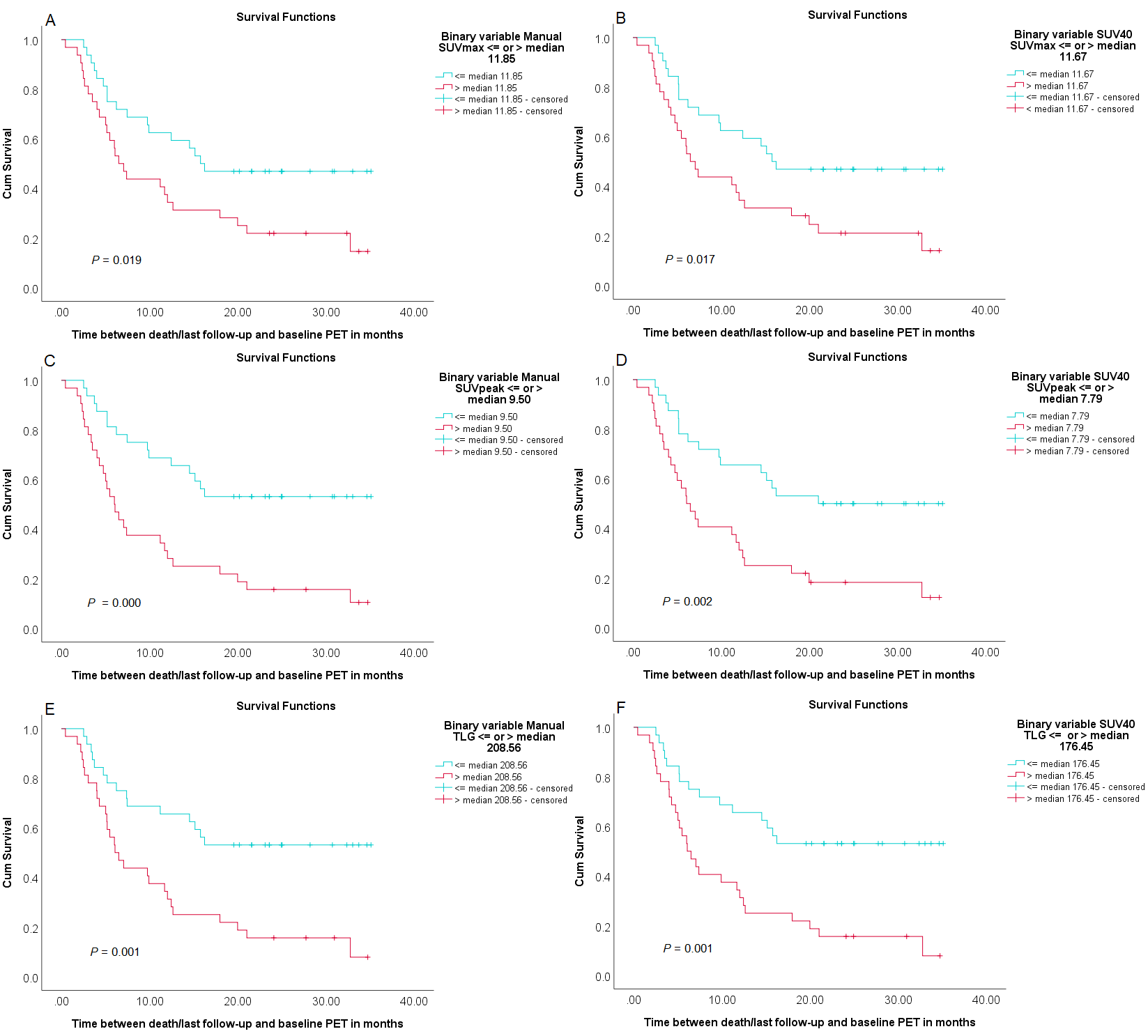
***

**Supplemental Fig. 1** Kaplan-Meier curves and log-rank test P values for overall survival of all patients (n = 64) based on median SUVmax, SUVpeak, and TLG obtained through manual segmentation (A, C, E) and semi-automatic segmentation using the semi-automatic SUV40 method (B, D, F; without additional lesion selection); SUV40 = the semi-automated segmentation method using a fixed SUV threshold of 4.0 g/mL


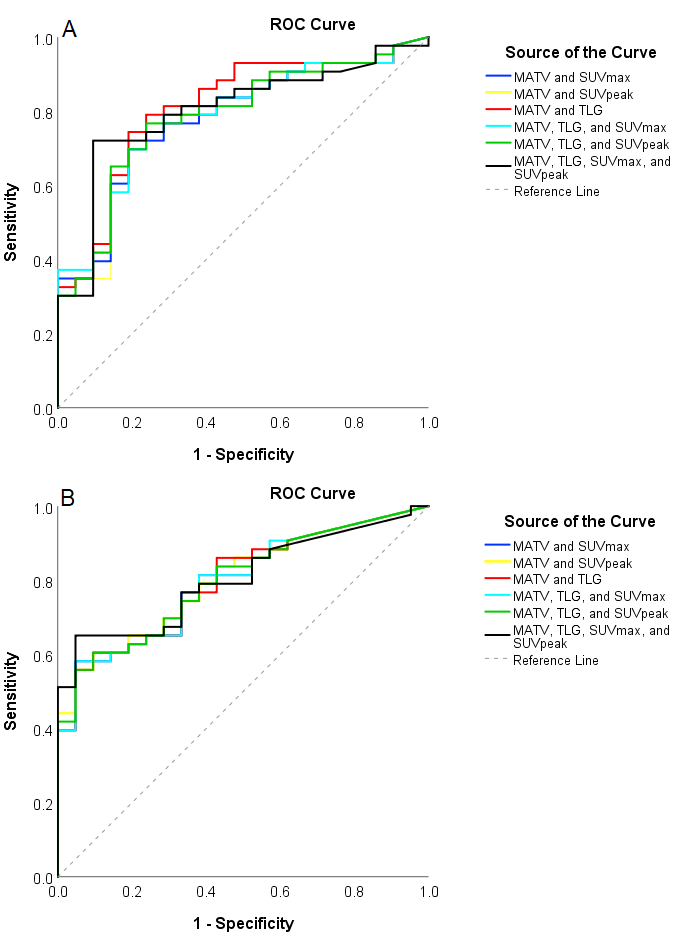


**Supplemental Fig 2** ROC curves assessing survival outcome based on combined MATV and SUVmax, SUVpeak, and TLG measurement by manual VOI segmentation (A) and VOI segmentation using the SUV40 semi-automatic segmentation method (B); SUV40 = the semi-automated segmentation method using a fixed SUV threshold of 4.0 g/mL

**Supplemental Table 1** Patient characteristics

| **Characteristic** | **All patients (*n* = 64)** |
| --- | --- |
| Age (years) at baseline PET/CT | 59 (45 – 69) (range 25 – 80) |
|  |  |
| Gender |  |
| Male | 40 (62.5 %) |
| Female | 24 (37.5 %) |
|  |  |
| Histology primary melanoma |  |
| Cutaneous | 47 (73.4 %) |
| Mucosal | 4 (6.3 %) |
| Primary melanoma unknown/missing | 13 (20.3 %) |
|  |  |
| M-stage at baseline PET/CT |  |
| M1a | 1 (1.6 %) |
| M1b | 2 (3.1 %) |
| M1c | 61 (95.3 %) |
|  |  |
| Organ involvement |  |
| (Sub)cutaneous | 39 (60.9 %) |
| Lymph nodes | 54 (84.4 %) |
| Lungs | 40 (62.5 %) |
| Muscular | 25 (39.1 %) |
| Skeletal | 39 (60.9 %) |
| Liver | 24 (37.5 %) |
| Abdomen | 30 (46.9 %) |
| Other | 14 (21.9 %) |

**Supplemental Table 2** Areas under the ROC curves (see Supplemental Fig. 2) for manual segmentation and VOI segmentation using the SUV40 semi-automatic segmentation method; SUV40 = the semi-automated segmentation method using a fixed SUV threshold of 4.0 g/mL

| Segmentation method and variable(s) | Area under the ROC curve |
| --- | --- |
| *Manual* |  |
| MATV | 0.806 |
| SUVmax | 0.720 |
| SUVpeak | 0.734 |
| TLG | 0.811 |
| MATV and SUVmax | 0.781 |
| MATV and SUVpeak | 0.782 |
| MATV and TLG | 0.810 |
| MATV, SUVmax, and TLG | 0.786 |
| MATV, SUVpeak, and TLG | 0.786 |
| MATV, SUVmax, SUVpeak, and TLG | 0.801 |
| *Semi-automatic*  MATV  SUVmax  SUVpeak  TLG  MATV and SUVmax  MATV and SUVpeak  MATV and TLG  MATV, SUVmax, and TLG  MATV, SUVpeak, and TLG  MATV, SUVmax, SUVpeak, and TLG | 0.790  0.733  0.762  0.811  0.794  0.801  0.796  0.794  0.797  0.804 |
